# Systematic Dysphagia Screening of Elderly Persons in the Emergency Department—A Feasibility Study

**DOI:** 10.3390/geriatrics5040075

**Published:** 2020-10-12

**Authors:** Dorte Melgaard, Line R. Sørensen, Diana Lund, Peter Leutscher, Marc Ludwig

**Affiliations:** 1 Centre for Clinical Research, North Denmark Regional Hospital, DK-9800 Hjoerring, Denmark; p.leutscher@rn.dk; 2 Department of Clinical Medicine, Aalborg University, DK-9000 Aalborg, Denmark; 3 Department of Emergency Medicine, North Denmark Regional Hospital, Bispensgade 37, DK-9800 Hjoerring, Denmark; lire@rn.dk (L.R.S.); marc.ludwig@rn.dk (M.L.); 4 Department of Physiotherapy and Occupational Therapy North Denmark Regional Hospital, Bispensgade 37, DK-9800 Hjoerring, Denmark; dpl@rn.dk

**Keywords:** swallowing disorder, geriatric patients, prevalence, screening, assessment

## Abstract

Identification of elderly patients in risk of dysphagia as early as possible upon hospital admission seems warranted due to the risk of aspiration pneumonia, dehydration, length of stay, and increased mortality. This study aimed to evaluate the feasibility and outcome of dysphagia screening of elderly persons admitted to the emergency department (ED). Inclusion criteria were age ≥ 60 years. A nurse applied the Simple Water Swallow test within one hour of admission. Subsequent assessment was performed by an occupational therapist (OT) using Volume Viscosity Swallow Test and Minimal Eating Observation Form. Of 113 eligible participants (median age 78 years), 75 (66%) were screened in the ED by the nurse, and among those, 12 (16%) were detected with dysphagia. Twenty of the patients not screened in the ED due to critical illness were tested by the OT in the ward after clinical stabilization and 15 patients (75%) were identified with dysphagia. This study demonstrated that it is feasible to perform dysphagia screening of elderly persons by a nurse in the ED, but there are severe limitations according to screening patients with critical illness and patients fasting before surgery in the ED. These patients have a high prevalence of dysphagia and should be screened as early as possible after hospitalization, as it will rarely be possible in the ED.

## 1. Introduction

Dysphagia is common in geriatric patients as well as in patients with neurological diseases, chronic obstructive pulmonary disease (COPD), and cancer [1,2,3]. It is well documented that dysphagia is associated with aspiration pneumonia, malnutrition, weight loss, frequent hospital admission with prolonged length of stay (LOS), increased mortality, and decreased quality of life [4,5]. Overall, dysphagia adds an extra burden in the management of the vulnerable patient groups with increased economic cost to the health care sector [6].

The goal of early and systematic screening for dysphagia is to identify patients in risk of dysphagia, thereby preventing the above-mentioned adverse effects of untreated dysphagia. When patients are screened for dysphagia at admission, the result allows for administrative consistency regarding food, liquids, and medicine to avoid aspiration pneumonia and to recommend rehabilitative interventions such as bolus modification and postural adjustment of the patient.

The positive effect of screening for dysphagia in patients with acute stroke is well known; a recent study indicates an effect of screening for dysphagia in an internal medicine unit, but there is no knowledge of the effects of early screening for dysphagia among patients admitted in the emergency department (ED) [7,8].

Instrumental assessment is recommended for assessment of dysphagia; the most common ways are fiberoptic endoscopic evaluation of swallowing and video fluoroscopy considered as the gold standard in diagnosing dysphagia [9].

These instrumental tests are, however, not relevant for use in an acute setting due to time constraints, need for trained personnel, and the patient’s condition.

Bedside screening is typically performed by use of a non-invasive test with the main purpose of detecting a tendency to aspirate when swallowing to identify patients with dysphagia for a further assessment and treatment. There are numerous bedside swallowing evaluation tests [10]. The most relevant requirements for a screening test are that it must be sensitive, specific, and easily administered without training. Moreover, the test also needs to be time- as well as cost-effective [11]. Water swallow test (WST) is commonly applied for dysphagia screening [11]. A validation study of WST documented a sensitivity of 96.5%, a specificity of 48.7%, and a false positive rate of 51.3% [12]. Dysphagia can also be assessed with other bedside tests, such as Volume Viscosity Swallow Test (V-VST) and Minimal Eating Observation Form-vers. II (MEOF-II), both of which are validated and recommended as assessment tools [11,13,14,15].

This feasibility study aimed to evaluate the logistic ability of using a simple water swallow test (WST) for early screening of dysphagia in patients ≥ 60 years admitted to an ED and to assess the outcome of dysphagia screening by use of WST among patients in the ED.

## 2. Materials and Methods

This cross-sectional study included hospitalized patients in the ED at the North Denmark Regional Hospital during the period of 27 February to 10 March in 2017, daily from 2 p.m. until 10 p.m.

Inclusion criteria was age ≥ 60 years. Fasting patients were excluded. Patients with acute stroke were admitted directly to the neurological ward, bypassing the ED.

There was a large spread in the causes of contact with the ED. The causes were categorized in following groups: neurological (e.g., Parkinson disease, stroke sequela, and dementia), circulatory (e.g., systemic arterial hypertension, congestive heart failure, diabetes mellitus, or acute myocardial infarction), respiratory (e.g., pneumonia, COPD, asthma), and others.

This is a quality-assurance study, and therefore, the Regional Ethical Committee of Northern Denmark waived the need for approval in February 2017. The study was registered with the Danish Data Protection Authority (2008-58-0028).

### 2.1. Dysphagia Screening by Water Swallow Test

Two nurses were trained to perform WST screening among the patients within one hour of admission to the ED. The WST consists of a test in which the patient is challenged by intake of volumes of 5 mL and 60 mL of water. Positive signs of potential dysphagia are cough and/or voice change when swallowing the water. The response combination of cough and voice change improves the predictive value of WST [16].

### 2.2. Confirmative Dysphagia Testing

Positive signs of dysphagia (cough and/or voice change by the WST) resulted in a referral to a trained occupational therapist (OT) who tested the patient using a confirmative test-battery, including V-VST and MEOF-II within 24 h after WST screening.

The V-VST assess swallow dysfunction by increasing volumes of 5 mL, 10 mL, and 20 mL, respectively, and the three viscosities water, nectar, and pudding, equaling a total of nine test scenarios [17]. The nectar viscosity was achieved by adding 1.2 g of the thickener Resource ThickenUp (Nestlé HealthCare Nutrition, Vevey, Schwitzerland) to 100 mL of mineral water, and pudding viscosity was achieved by adding 6.0 g of the same thickener. Boluses of each volume and viscosity were offered to the patients with a syringe during the test to ensure an accurate measurement of the volume. During testing, the oxygen saturation was measured contemporarily with a pulse oximeter and the following clinical signs of impaired efficacy of swallowing were observed: impaired labial seal, oral or pharyngeal residue, and multiple swallows per bolus. According to V-VST, the following clinical signs of impaired safety of swallowing were also observed: changes of voice quality, cough, or decrease in oxygen saturation ≥ 3% to detect silent aspiration. One sign or more of impaired safety or efficacy indicated the presence of dysphagia [17].

The MEOF-II was used as a complementary assessment tool. MEOF-II includes nine items in three subscales: ingestion (sitting position, manipulation of food on the plate, transport of food to the mouth), deglutition (manipulation of food in the mouth, swallowing, or difficulties in chewing); energy and appetite (eats less than 3/4 of served portion, energy, or appetite). For each item, a rating of zero indicates normal eating and one indicates eating difficulty. The chewing ability with a score of seldom or never having problems was dichotomized as zero, and a score with very often, quite often, now and then, or occasionally having problems was scored with one. Appetite was dichotomized as zero when scored with strongly increased, increased, or normal, or one when scored with reduced or strongly reduced [18].

### 2.3. Statistical Analysis

Descriptive statistics for the demographic variables include the number and percentage of patients for categorical variables, and continuous variables were summarized as their median (5th percentile; 95th percentile).

## 3. Results

A total of 113 patients (56% male, median age 78 years (62; 93)) were eligible for participation in the study and among those 75 (66%) were screened for dysphagia in the ED.

The results of the screening presumed a prevalence of dysphagia in the selected study population of 16% (*n* = 12 patient). The positive screening results from the WST were confirmed with V-VST and MEOF-II in all 12 patients with dysphagia. Table 1 illustrates the characteristics of the entire group of patients, patients with signs of dysphagia, patients with no signs of dysphagia, and patients not screened in the ED. The groups are comparable according to gender and age, but there is a tendency for a higher prevalence of dysphagia in patients contacting the ED because of circulatory and respiratory diseases.

As illustrated in Table 1 patients screened positive for dysphagia have a higher incidence of COPD, neurological diseases and diabetes as well as more of the patients with dysphagia have had an unintended weight loss compared to patients with no dysphagia.

A total of 38 patients (50% male, median age 79 years (63; 93)) were not screened in the ED, due to the following reasons: 26 patients (68%) due to poor health conditions and need for acute care, nine patients (24%) due to limited time, and 3 patients (8%) declined participation.

Out of the 26 patients (54% male, median age 81 years (62; 94)) not screened due to poor health conditions in the ED, 20 patients (50% male, median age 83 years (66; 95)) were tested in the hospital ward by an OT with the V-VST and MEOF-II. Fifteen (75%) (53% male, median age 82 years (76; 91)) of the 20 patients not screened in the ED were found with dysphagia when tested afterwards in the ward. Reasons for hospitalization in these 15 patients tested positive for dysphagia were respiratory diseases (*n* = 6, 40%), neurological diseases (*n* = 4, 27%), circulatory diseases (*n* = 3, 20%), and other (*n* = 2, 13%). In this group of patients, six (40%) patients were known with neurological disease, one (7%) patient with COPD, and one (7%) patient with unintended weight loss.

## 4. Discussion

The present study documented a presumed prevalence of dysphagia of 16% in patients who were screened less than one hour after they were admitted to the ED. In the group of poor health condition and in need for acute care an assessment for dysphagia within 24 h demonstrated a prevalence of 75%.

In many hospitals, nurse-initiated dysphagia screening for patients with neurological dysfunction is a common practice [19,20], and reduction in time to swallow, fewer incidents with chest infections, and lower mortality are beneficial outcomes of systematic screening in this group of patients with acute stroke. [7,21]. To our knowledge, no studies have evaluated the applicability and outcome of dysphagia screening as early as in the ED.

The WST was chosen as screening tool even though it has a low specificity and a high false positive rate, because, on the other hand, the sensitivity is documented to be high to identify patients at risk of aspiration, combined with another relevant factor, in that the WST is very time-effective. Other screening tools could have been chosen e.g., the 10-item Eating Assessment Tool (EAT-10) which is a self-administered, symptom-specific outcome instrument for dysphagia, but it has some psychometric limitations and a main issue is that patients must be linguistically and cognitively intact to fulfill the form [22,23,24]. Alternatively, the Gugging Swallowing Screen (GUSS) typically used as a screening tool for acute stroke patients could be used, but it has limitations as well, according to the acute inpatient population [25,26]. Recently, a new tool consisting of four questions has been introduced, but it was not available when the present study was performed [27]. The V-VST could be an alternative as well, as it can be used as a screening or assessment tool depending the testers’ qualifications and the setting. In the ED setting, it was decided that it was too time-consuming, as well as the V-VST requires more training of the nurses than the WST. When patients were screened positive with the WST, it was confirmed by a trained OT using the V-VST and MEOF-II to assess for dysphagia in the patients. The results showed high sensitivity, confirming the study of Suiter et al. and Brodsky et al. [16]. In the present study, in the ED it was not possible to make instrumental studies of dysphagia, despite it being recommended as the golden standard [28].

In the present study, patients fasting before surgery were excluded. This limitation is important due to the fact that it is well documented that the prevalence in these groups of patients is up to 60% [3,29,30,31]. This indicates a need for screening for dysphagia in this group of patients within hours after operation in order to reduce the risk of lung infection, which can lead to complications and prolonged hospitalization.

If screening is not possible due to a very short stay in the ED, then the screening must be standard in the ward. The focus in screening the patients very early for dysphagia after admission is to prevent the effects of untreated dysphagia during the hospitalization. In this study, patients were not retested before discharge, but in a future study it will be relevant to uncover whether the condition is temporary and related to e.g., an untreated infection or the patient still having dysphagia when they are discharged.

The present study suggests a clinically feasible possibility of screening of patients in the ED. In this study, two nurses were trained in screening with the WST. For the implementation of the screening for dysphagia, a guideline will be needed [21].

Additional larger studies are needed to detect whether early screening for dysphagia in patients with severe lung or respiratory diseases, geriatric patients, and hip fracture patients in the ED or within a few hours after admission will influence the treatment and reduce the frequency of aspiration pneumonias and LOS in hospital. A future larger study may also provide knowledge about predictors for dysphagia in the ED, and then a future screening can be focused.

## 5. Conclusions

This study documents that it is possible to use the WST as a screening tool in the ED. It is well clarified that a significant group of patients cannot be screened in ED either due to severe conditions or because they fast before surgery. To be able to focus a future screening for dysphagia in the ED and the acute setting, it is necessary to provide knowledge about predictors for dysphagia in the ED.

These groups need an increased focus and should be screened within a few hours after admission or as soon as possible to prevent aspiration pneumonias.

We suggest that the present study leads to a well-powered multicenter randomized controlled study for developing evidence for screening for dysphagia in the ED.

## Figures and Tables

**Table 1:** Demographical and clinical characteristics of patients.

	All Patients *n* = 113	Dysphagia *n* = 12	Not Dysphagia *n* = 63	Not Screened *n* = 38
Gender	-	-	-	-
Male	63 (56%)	6 (50%)	38 (60%)	19 (50%)
Female	50 (44%)	6 (50%)	25 (40%)	19 (50%)
Age, year	78 (62; 93)	78 (61; 94)	76 (61; 93)	79 (63; 93)
ED contact categories	-	-	-	-
Neurological	18 (16%)	1 (8%)	9 (14%)	8 (21%)
Circulatory	29 (26%)	5 (42%)	17 (27%)	7 (18%)
Respiratory	49 (43%)	6 (50%)	25 (40%)	18 (47%)
Other	17 (15%)	0 (0%)	12 (19%)	5 (13%)
Comorbidities	-	-	-	-
COPD	22 (20%)	4 (33%)	9 (14%)	9 (24%)
Neurological disease	20 (18%)	3 (25%)	7 (11%)	10 (26%)
Unintended weight loss	15 (13%)	4 (33%)	8 (13%)	3 (8%)
Diabetes	10 (9%)	2 (17%)	7 (11%)	1 (3%)
Alcoholism	4 (4%)	0 (0%)	2 (3%)	2 (5%)

Data are represented as number of patients (%) and median (5th percentile; 95th percentile). Chronic Obstructive Pulmonary Disease (COPD); Emergency Department (ED).
